# Risk Factors and Therapeutic Targets in Pancreatic Cancer

**DOI:** 10.3389/fonc.2013.00282

**Published:** 2013-11-18

**Authors:** Sonja Maria Wörmann, Hana Algül

**Affiliations:** ^1^II. Medizinische Klinik, Klinikum rechts der Isar, Technische Universität München, Munich, Germany

**Keywords:** pancreatic ductal adenocarcinoma, risk factors, hereditary cancer syndromes, therapeutic targets, signal-transduction pathways, immune response, stroma reaction, epigenetic changes

## Abstract

Pancreatic cancer (PC) is one of the most challenging tumor entities worldwide, characterized as a highly aggressive disease with dismal overall prognosis and an incidence rate equalling mortality rate. Over the last decade, substantial progress has been made to define the morphological changes and key genetic events in pancreatic carcinogenesis. And yet, it is still unclear what factors trigger PC. Some risk factors appear to be associated with sex, age, race/ethnicity, or other rare genetic conditions. Additionally, modifying factors such as smoking, obesity, diabetes, occupational risk factors, etc., increase the potential for acquiring genetic mutations that may result in PC. Another hallmark of PC is its poor response to radio- and chemo-therapy. Current chemotherapeutic regimens could not provide substantial survival benefit with a clear increase in overall survival. Recently, several new approaches to significantly improve the clinical outcome of PC have been described involving downstream signaling cascades desmoplasia and stromal response as well as tumor microenvironment, immune response, vasculature, and angiogenesis. This review summarizes major risk factors for PC and tries to illuminate relevant targets considerable for new therapeutic approaches.

## Introduction and Facts

Pancreatic cancer (PC) is one of the most challenging tumor entities worldwide, characterized as a highly aggressive disease with dismal overall prognosis and an incidence rate (IR) equaling mortality rate (MR). Although over the past decade a downward trend in rates for most other major cancer sites could be observed, PC shows raising incidence and unfavorable MRs among men and women overall (1, 2). PC represents the eight leading cause of cancer-related death worldwide accounting for 4% of all cancers with approximately 266,669 deaths, out of 278,684 new cases in 2008 for both sexes (3–6), and displays the fourth-leading cause of cancer-related death in the U.S. (7), and the E.U. (1) among both men and women. Importantly, MRs are highest in more high-income areas of the world, intermediate in South and Central America and eastern Asia, and lowest in low-income areas (6, 8). Despite advances in surgery, chemotherapy, and radiation therapy, the prognosis of PC remains extremely poor with a 1- to 5-year overall survival rate about 25–6%, which is the lowest 5-year overall survival rate of any cancer in the U.S. (9), and a 5-year survival rate <10% in the E.U. (8). Prognosis of PC is largely determined by the stage (TNM classification) of disease at diagnosis. Between 2002 and 2008, around 8–9% of all U.S. PC patients were diagnosed with local disease, 25–27% with regional disease, and 52–56% with distant disease (10, 11). The median survival ranges from 4.5 months for the most advanced stage to 24.1 months for the earliest stage (9, 12). Due to the fact, that PC has a poor response to radio- and chemo-therapy (13), surgery provides at present the only potentially curative treatment prolonging survival. However, <15% (14) of all PC patients are candidates for surgical resection after which 5-year survival rarely exceeds 20–25% (15). The 5-year survival time for local disease is 24.1%, 9% for regional, 2% for distant disease, and the overall survival for the average patient reaches only 6% (10). The exact causes of PC are not known but some factors such as sex, age, race/ethnicity appear to be associated with PC. Additionally, modifying factors such as smoking, obesity, diabetes, occupational risk factors, etc., increase the potential for acquiring genetic mutations that may result in PC (Table 1).

**Table 1:** **Risk factors for PC**.

Risk types	Factor dependent risk levels for PC	Reference
**ESTABILSHED/FIXED RISK**		
Sexes	PC is about 30% more common in men than in women	American Cancer Society (ACS) (9), Howlader et al. (10)
Socioeconomic status	Low-income correlates with 80% increased PC risk in white man and 170% in African American men	Silverman et al. (16)
Age	Advanced age is an important risk factors for increasing PC incidence and mortality rates	World Health Organization (2), Ferlay et al. (5), American Cancer Society (ACS) (9)
Race/ethnicity	Incidence and mortality rates of PC were found highest in African Americans, intermediate in white Americans and lowest rates in Asian Americans/Pacific Islanders	American Cancer Society (ACS) (9), Silverman et al. (16)
**MODIFIABLE RISK**		
Smoking	Smoking is responsible for 20–30% of PC. PC risk increases at 74%	Parkin (17), Iodice et al. (18), Parkin et al. (19)
Obesity and physical activity	Risk is 20% higher for obese individuals; high waist-to-hip ratio increases risk independently of general obesity	Berrington de Gonzalez et al. (20), Arslan et al. (21), Aune et al. (22), Luo et al. (23)
Alcohol use	Studies are inconsistent: first three or more drinks of alcohol per day increased risk at 20–30%; in contrast: second no increased risk for consumption of 60 g/day or more of liquor and no association with beer or wine	Tramacere et al. (24), Michaud et al. (25)
Dietary factors	Red and processed meat slightly increases risk; conflicting studies about meat containing high mutagen levels and PC risk; arguable protective effect for folate intake; most likely reduced risk due to fruits and vegetables consumption; no risk correlation for intake of coffee or tea and PC; increased PC risk for sugar-sweetened carbonated soft drink intake	Larsson and Wolk (26), Anderson et al. (27), Jansen et al. (28, 29, 30), Larsson et al. (31), Bao et al. (32), Vrieling et al. (33), Koushik et al. (34), Turati et al. (35), Genkinger et al. (36)
Vitamin D	Inconsistent studies: vitamin D is likely to be protective. No correlation of low levels of vitamin D and PC, but twofold increased risk for high vitamin D levels were found recently	Grant (37), Boscoe and Schymura (38), Mohr et al. (39), Bao et al. (40), Giovannucci (41), Stolzenberg-Solomon et al. (42, 43)
Diabetes	Long-term diabetes type II increases PC risk at 50%. PC risk is increased for diabetes independent on duration, for hyperglycemia, abnormal glucose metabolism, insulin resistance, and for type I diabetes	Huxley et al. (44), Henry et al. (45), Jee et al. (46), Stolzenberg-Solomon et al. (47), Stattin et al. (48), Stocks et al. (49), Stevens et al. (50)
**OTHER RISK FACTORS**		
Infection and other medical conditions	Chronic infections with hepatitis B virus, hepatitis C virus, *Helicobacter pylori*, history of cholecystectomy or partial gastrectomy, cystic fibrosis, periodontal disease, and blood groups A, AB, and B increase risk for PC	Hassan et al. (51), El-Serag et al. (52), Risch et al. (53), Lin et al. (54), Gong et al. (55), Maisonneuve et al. (56), Fitzpatrick and Katz (57), Wolpin et al. (58), Pelzer et al. (59)
Chronic pancreatitis	Six-fold increased risk due to chronic pancreatitis; risk correlates with duration of recurrent pancreatitis and chronic inflammation. Life-time risk of PC in hereditary pancreatitis is 40%; only 4% of chronic pancreatitis patient develop PC within 20 years	Raimondi et al. (60), Whitcomb (61), Lowenfels et al. (62, 63)
**GENETIC RISK**		
Family history	Around 10% of PC are referable to inherited genetic factors	Petersen et al. (64), Shi et al. (65)
	Life-time risk for PC is 1.3–1.5% in general population; for individuals with a family history of PC increased risk of to two- to threefold; risk is around 6.4-fold greater in individuals with two FDRs and 32-fold greater in individuals with three or more FDRs	American Cancer Society (ACS) (9), Brune et al. (66), Lynch et al. (67), Silverman et al. (68), Tersmette et al. (69), Klein et al. (70), Canto et al. (71), Wang et al. (72), Grover and Syngal (73)
Hereditary cancer syndromes	Hereditary breast and ovarian cancer (*BRCA1*/*BRCA2*): RR: 3.5 (BRCA2); RR 2.3 (BRCA1)	Couch et al. (74), Hahn et al. (75), Murphy (76)
	The Peutz–Jeghers syndrome (*STK11*/*LKB1*): PC life-time-risk: 11–36%	van Lier et al. (77), Giardiello et al. (78), Korsse et al. (79)
	Familial-atypical multiple mole melanoma syndrome (*CDKN2A*): cumulative PC risk 17%	Lynch et al. (80), de Snoo et al. (81), Vasen et al. (82)
	Li–Fraumeni (*TP53*): PC RR: 7.3	Birch et al. (83), Kleihues et al. (84), Ruijs et al. (85)
	Hereditary non-polyposis colorectal cancer (*MLH1, MSH2, MSH6, PMS2*): PC life-time-risk: 3.7%	Shi et al. (65), Win et al. (86), Kastrinos et al. (87)
	Familial adenomtosis polyposis (*APC*): PC RR 4.46	Giardiello et al. (88)
	Ataxia teleangiectasia (*ATM*): PC RR 2.41	Roberts et al. (89), Bakker and de Winter (90)
Hereditary syndromes with chronic inflammation/dysfunction of gland	Hereditary pancreatitis (*PRSS1, SPINK1, PRSS2, CTRC*): PC cumulative risk: 40–55% Cystic fibrosis (*CFTR*): PC RR: 5.3	Raimondi et al. (60), Rebours et al. (91), Martin and Ulrich (92), Lowenfels et al. (93) Maisonneuve et al. (56), McWilliams et al. (94)
Other causative germ-line mutation for FPC	*PALB2* in 4.9% of FPC	Schneider et al. (95), Slater et al. (96), Harinck et al. (97)
	*BCRA2* in 3–17% of FPC	Schneider et al. (95), Hahn et al. (75), Murphy et al. (76), Slater et al. (98)
	*PALLD* seldom in FPC	Pogue-Geile et al. (99), Slater et al. (100)

### Established risks

#### Sexes

Pancreatic cancer is about 30% more common in men than in women. Data from U.S. (9, 10), E.U., and worldwide (4, 101) demonstrate higher incidence and MRs (ASR per 100,000 persons of 13.6/12.5) for men compared to women (ASR per 100,000 persons of 10.5/9.5). The observed gender specific disparity might be attributed to differential lifestyle habits such as use of tobacco, alcohol, etc., hormone dependence has not been confirmed so far (102).

#### Socioeconomic status

Interestingly, socioeconomic status measured as years of education negatively correlates with PC mortality (103). In addition, low income was associated with an 80% increased PC risk in white man along with an even higher increased risk of 170% in African American men after accounting for differences in heavy alcohol drinking, smoking, and dietary factors (16).

#### Age

Advanced age is one of the most important risk factors for increasing PC incidence and MRs. Notably, the risk is low until the age of 50, but steeply increases after that. Retrospectively, PC IRs in 35- to 39-year-old men increased from 1.2 up to 100.5 among subjects reaching 85 years and more. Similar findings could be observed in women, with increasing IRs from 1.0 (35–39 years of age) to 87.7 (over 85 years of age) (2, 5, 9). The median age of PC is diagnosed at 71 years (9) in the U.S. and 72 years in the U.K. (17), meaning that about half of all patients develop the disease at an age higher than 71/72 years. An even greater risk was found in patients with familial PC as the average age at diagnosis in these individuals is around 68 (66).

#### Race/ethnicity

The IRs and MRs of PC vary across different ethnic groups. In the U.S. the highest rates were found in African Americans (IR:15.3/MR: 13.8), followed by intermediate rates in white Americans (IR:11.6/MR:10.7), and lowest rates in Asian Americans/Pacific Islanders (IR: 8.8/MR: 7.5; data respectively for 2005–2009) (9). Commonly, IRs are higher in African Americans than in white Americans at every age. The racial disparity might be almost entirely explained by factors such as cigarette smoking and long-term diabetes mellitus for men and by moderate/heavy alcohol consumption and elevated body mass index for woman (16).

### Modifiable risks

#### Smoking

Smoking is the most important risk factor for PC and estimated to be responsible for approximately 20–30% of PC (17–19). A dose- and duration-related risk increase contributing to an earlier age of PC onset was found in several studies (104–106). Smokers face a 74% higher risk for PC compared to non-smokers (107). A European-wide study in 2012 provided evidence for a 27% risk increase for every five cigarettes smoked per day (108). Interestingly, several studies showed evidence that the deleterious effects of alcohol and tobacco in PC occurrence and mortality appear to resolve after 10 years of abstinence (18, 106, 109). For smokers, the risk can even be reduced to levels of non-smokers after 5 years of cessation as assessed by a European-wide prospective study in 2009 (109). Smokers with a family history of PC have an even greater risk of developing PC than non-smokers (66). For secondhand smoke exposure and PC the European (EPIC) study showed that passive smoking can increase the risk of PC by 50% (109) and children who are exposed daily to tobacco smoke have double the risk of developing PC later in life (110). In addition, cigar and pipe smoking (18, 111) as well as the use of smokeless tobacco (112) and moist snuff (113) have also been associated with increased PC risk (114).

#### Obesity and physical activity

Several studies have linked obesity and overweight to increased PC risk. The risk for developing PC was found to be around 20% higher for obese compared to normal weight individuals (20, 21). An estimated 12% of all PC cases in the UK are attributed to overweight and obesity (17, 19). In both men and women a BMI of 25 was associated with an increased risk for PC, but that risk was more pronounced in humans with a BMI of 35 or greater with an overall increase by 10% per five-point increase in BMI (22). Interestingly, having a high waist-to-hip ratio increases PC risk independently of general obesity (21, 22). Specifically, a 70% increase in PC risk for women having a high waist-to-hip ratio was assessed (23). Moreover, overweight and obesity during early adulthood where not only associated with a greater risk but also earlier disease onset of PC. Thereby, obesity at an older age was correlated to a lower overall survival in patients with PC (115). The mechanisms behind these observations are not well understood, however chronic inflammation, mediated by molecules such as TNFα and IL-6, feedback loops associated with the obese state, and disruptions in autophagy promoting ER stress and mitochondrial dysfunction are likely to be involved (116). Supporting this hypothesis, a study suggested that dietary and other lifestyle factors influencing insulin resistance are associated with PC risk (117). At present, there is no well-defined evidence correlating physical activity and PC risk (118, 119). More recently, recreational physical activity was proposed to exert a protective effect on PC risk (120), however this was not confirmed by a previous study (121).

#### Alcohol use

There is no clear evidence whether alcohol use causes PC or not. In several but not all previously performed studies alcohol use was correlated to increased risk for developing PC. A modest increase in PC risk was observed with consumption of 30 or more grams of alcohol per day (122). A meta-analysis found that consumption of three or more drinks of alcohol per day was associated with a 20–30% increased risk of PC (24). However, a recent large nested case-control study in 2010 showed no increased risk, even at consumption of 60 g/day or more of liquor, and found no association with beer or wine (25).

#### Dietary factors

A number of dietary factors have been assessed regarding their association with PC risk. There is some evidence that consumption of red and processed meat may slightly increase PC risk (26). However, studies are conflicting about meat containing high mutagen levels and increased risk of PC (27, 28). A protective effect has been reported previously for folate intake (29), although it could not be confirmed in a recent large analysis (30). At present, opposing evidence exists regarding the effect of fruit and vegetable consumption on PC risk (31–33). A recent study suggests, that most nutrients obtained through consumption of fruits and vegetables may reduce the risk of developing PC (34). Overall, no association was observed for intake of coffee or tea and PC (35, 36). Interestingly, a positive association for risk of PC was found in the case of sugar-sweetened carbonated soft drink intake raising insulin and glucose levels, and thus promoting obesity and diabetes (36, 123–125).

#### Sunlight and vitamin D

Results correlating vitamin D from sun exposure and PC are inconsistent. Some studies associated sun exposure with reduced PC death rates, suggesting that vitamin D might protect against PC (37–39). Other studies also assumed that dietary vitamin D and vitamin D derived from both diet and sunlight exposure might be protective (40, 41). In an additional study, no positive association between 25(OH)D and PC was found, although the previous finding correlating increased PC risk with low residential UVB exposure could be confirmed (42). However, more recently, no correlation of low levels of vitamin D and PC was found and it was even suggested that high vitamin D levels are associated with twofold increased risk of PC risk (43).

#### Diabetes

Diabetes mellitus is both risk factor for PC and as new onset diabetes a potential early disease sign (126). At diagnosis about 25% of PC patient suffer from diabetes mellitus another 40% are pre-diabetic (126, 127). Patients with long-term (≥5 years) diabetes type II have a 50% increased risk of PC compared with non-diabetic individuals (44). Recently performed studies provide evidence for elevated PC risk with diabetes independent on duration (45) and suggest that hyperglycemia, abnormal glucose metabolism, and insulin resistance correlate with increased risk of PC (46–49). Increased PC risk has also been reported among individuals with type I diabetes (50). Moreover, T3cDM referred to as secondary diabetes was evidenced as major subset of diabetes potentially accounting for the highest risk of PC in particular in patients with chronic pancreatitis. Further, T3cDM was pronounced as consequence of PC in at least 30% of patients (128). Additional preliminary data suggest, that GCKR rs780094, a single-nucleotide polymorphism related to diabetes, is associated with PC risk (129).

### Other risk factors

#### Infections and other medical conditions

Several studies demonstrated increased PC risk among people with chronic hepatitis B (51), hepatitis C (52), and *Helicobacter pylori* (53) infections. In addition, a history of cholecystectomy (54) or partial gastrectomy (55) as well as other medical conditions including cystic fibrosis (56) and periodontal disease (57) were associated with increased PC risk. Recent studies suggested a slightly increased risk of PC for people with non-O blood groups (i.e., blood groups A, AB, and B) whereas blood group O was less frequent in patients with PC. However, the mechanisms behind this carcinogenesis association are still unclear (58, 59).

#### Pancreatitis

Several studies provide evidence for a strong association between long-standing chronic pancreatitis and PC. Importantly, pancreatitis is also considered an early indicator of PC (60, 130). An even sixfold increased risk of PC among patients with chronic pancreatitis after excluding PC was reported in a review study (60). The risk correlates with the duration of recurrent pancreatitis and chronic inflammation (61). An even higher risk was found in patients with rare types of pancreatitis, such as hereditary pancreatitis and tropical pancreatitis. The assessed life-time risk of PC in individuals with hereditary pancreatitis is with about 40% high, reaching approximately 75% in paternal inheritance pattern (62). The lag of time between pancreatitis diagnosis and PC onset is usually about 10–20 years. Despite these positive correlations chronic pancreatitis as risk for PC is still uncommon as only about 4% of chronic pancreatitis patient will develop PC within 20 years of diagnosis (63).

### Genetic risk

Although the majority of PC appears to be sporadic, around 10% of PC cases are attributable to inherited genetic factors (64, 65). Inherited predisposition to PC is currently classified in three distinct clinical settings: first, tumor predisposition syndromes such as hereditary breast and ovarian cancer, Peutz–Jeghers Syndrome (PJS), familial-atypical multiple mole melanoma (FAMMM), pancreatic-melanoma cancer syndrome (PCMS), or Li–Fraumeni-syndrome, etc., which are characterized by a clinical phenotype other than PC, but known to correlate with an increased risk of PC; second, hereditary pancreatitis and cystic fibrosis, in which genetically determined early changes of the pancreas might predispose to the development of PC; third, familial pancreatic cancer (FPC) which refers to families with two or more first-degree relatives (FDRs) with PC that do not fulfill the criteria for another inherited predisposition syndrome (95, 131, 132). The term FPC is used in the case of three or more relatives of any degree (133).

#### Family history

Accumulating evidence links family history with PC (67–70). Based on strict inclusion criteria a familial aggregation of PC was reported to be only 2.7 and 1.9% in two prospective studies from Sweden and Germany, respectively (134, 135). The life-time PC risk of 1.3–1.5% in the general population is low (9, 71), but for individuals with a family history of PC the risk can increase dramatically two- to threefold (71). Thereby, the risk stratification depends on the number of affected family members and the relationships between at risk individuals (70, 72). The risk has been estimated to be 6.4-fold greater in individuals with two FDRs with PC (life-time risk 8–12%) (73) and 32-fold greater in individuals with three or more FDRs with PC (life-time risk 40%) (66, 70, 73). Moreover, the risk is increased if a FDR is diagnosed with PC before age 50 (66).

#### Hereditary cancer syndromes, genetically determined early changes of the pancreas and FPC

Interestingly, PC occurs frequently in excess of expectance in several hereditary cancer syndromes, which are associated with specific germ-line gene mutations. These syndromes show an increased risk for PC varying from 5 up to 40% (136). Clinically defined familial cancer syndromes are extremely rare in the general population accounting only for a small number of FPC (112, 137). For example: familial breast cancer mutations in the *BRCA2* gene, associated with a 3- to 10-fold increased risk of PC, account for increasing frequency and the highest proportion (5–17%) of known causes of inherited PC (74–76). A relative risk (RR) of 3.5 (95% CI 1.87–6.58) for PC in *BCRA2* gene mutation carriers was observed (138, 139), whereas for *BRCA1* gene mutation only a modestly increased RR of 2.3 for PC was demonstrated by a cohort study (140). More recently, an approximately doubled risk for PC was found in female *BRCA* carriers with a standardized IR for *BRCA1* of 2.55 and for *BRCA2* of 2.13 (141). The PJS, usually caused by germ-line mutations in the *STK11*/*LKB1* gene, has a 132-fold increased risk for developing PC and shows a cumulative life-time-risk of 11–36% up to age 65–70 among affected individuals (71, 77, 78). A recent study proposed a cumulative risk for PC in individuals with PJS of 26% at age 70 years and a RR of 76 (95% CI 36–160) (79). Hereditary non-polyposis colorectal cancer (HNPCC or Lynch syndrome), associated with mismatch repair genes (*MLH1, MSH2, MSH6, PMS2*) mutations (132) has an estimated life-time risk of 3.7% for developing PC (8.6-fold higher risk) (65, 86, 142). FAMMM-PC syndrome, an autosomal dominant disease with variable penetrance and linked to mutations in the *CDKN2A* tumor-suppressor gene (143, 144), is associated with an approximately 13- to 22-fold increased risk of PC (80, 81). The estimated cumulative risk of developing PC in putative mutation carriers by age 75 years was 17% (82). Despite extensive study, germ-line *p16* mutations in PC have not been found in the absence of any manifestation of familial-atypical multiple molmelanoma. More recently, Roberts et al. showed in a cohort of 166 familial PC probands that at least 2.4% (4/166) of familial pancreatic cases could be explained by deleterious ataxia-telangiectasia (*ATM*) mutations (89). Thus, the risk for PC was found to be increased with a RR of 2.41 (95% CI 0.34–17.1) (90). Familial adenomatous polyposis (FAP) linked to mutations in the *APC* tumor suppressor gene has also been associated with an increased risk for PC. A RR of 4.46 (95% CL 1.2–11.4) in polyposis patients and at risk relatives could be observed (88). Further, the Li–Fraumeni syndrome, a cancer predisposition syndrome featuring germ-line mutation of the *p53* tumor suppressor gene, is characterized by a high incidence of a variety of cancers diagnosed at young ages. PC seems to be moderately associated with Li–Fraumeni (83) as only 1.3% of all cancers in Li–Fraumeni patients are PC (84). The expected risk of developing PC for *p53* mutation carriers compared to the general population is increased with a RR of 7.3 (85). Another inherited genetic risk correlates with cystic fibrosis, characterized by mutations in the cystic fibrosis transmembrane conductance regulator (*CFTR*) gene which is associated with chronic idiopathic pancreatitis (145). A twofold increased risk for PC before the age of 60 years (94) and a RR of 5.3 (95% CI 2.4–10.1) for patients with *CFTR* carrier status was proposed (56). Hereditary pancreatitis associated with mutations in the cationic trypsinogen gene, *PRSS1* (143), *SPINK1, PRSS2*, and *CTRC*, also lead to an increased risk ranging between 26- and 70-fold compared to the general population, with a cumulative risk of 40–55% by age 70 for developing PC (60, 91–93). Aside from the previously discussed hereditary cancer syndromes and genetically determined early changes of the pancreas, which are not accounting for many cases of FPC, only few other causative germ-line mutation have yet been reported in FPC: *BRCA2* mutations were found to be causative for 15% of FPC in the EUROPAC study, even in the absence of breast cancer (75), for 17% of FPC as reported by the Hopkins group (76) and for 3% of FPC as proposed by the FaPaCa study (95). Interestingly, unclassified variants of *BRCA2* mutations of unknown clinical importance were detected in 8.6% of cases in the FaPaCa study (98). In addition, the BRCA2-interacting protein PALB2 was identified as a PC susceptibility gene (146) with germ-line mutations found in up to 5% of patients with FPC (95–97). Further, oncogenic mutation P239S in the Palladin (*PALLD*) gene, found in a linkage analysis of one large FPC family, has been proposed to be an additional major PC susceptibility gene (99). However, in the analysis from EUROPAC and FaPaCa families neither linkage nor any *PALLD* mutation was found (100). For other tumor suppressor genes such as *MAP2K4, MADH4* (*SMAD4*/*DAC4*), *ACVR1B* (*ALK4, activin receptor type 1B*) known to undergo germ-line or somatic genetic inactivation in clinically sporadic PC, no germ-line mutations could be identified in any of the FPC kindreds tested (76). Despite, previous linkage to PC none or non-deleterious germ-line mutations in *RNASEL* (147), *STK11* (148), *CHEK2* (149), and *NOD2* (150) genes could be found in the FaPaCa collection study (147–150). Additionally, *CDKN2a* mutations could only be detected in PCMS or FAMMM-PC families (151), as none of the *CDKN2a* mutations were identified in FPC families without melanoma (98, 152). In summary, only *BRCA2, PALB2*, and *PALLD* germ-line mutations could be observed in FPC families, potentially predisposing to PC. Importantly, the major gene (s) responsible for the inheritance patterns of PC remain to be identified.

## Potential Therapeutic Targets

**Figure 1:**
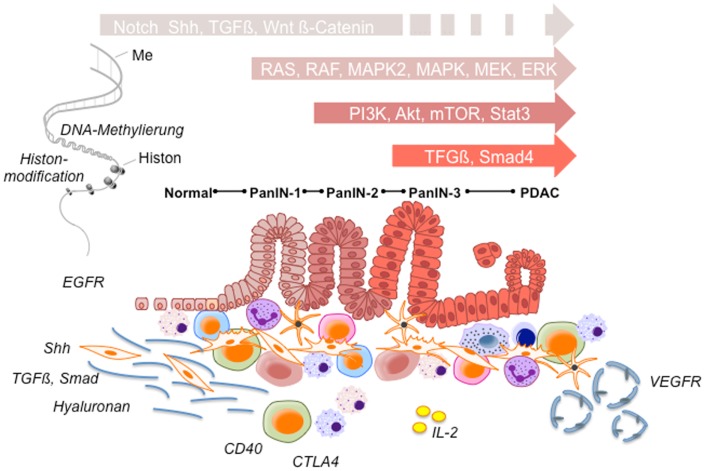
**Potential therapeutic targets**. During pancreatic cancer development a variety of signaling pathways participate in multiple stages of pancreatic tumorigenesis from early precursor lesions, histologically defined as pancreatic intraepithelial neoplastic lesions (PanINs 1–3 lesions) to advanced ductal pancreatic cancer. These histopathological changes are accompanied by infiltrating immune cells and an increasing desmoplastic stromal response. According to significantly involved signaling pathways tumor cell survival, angiogenesis, invasion, desmoplasia, and tumor immune response are affected, respectively. Ensuing alterations together with epigenetic changes are strongly involved in promoting tumor progression and chemotherapy resistance, and thus provide potential therapeutic targets in pancreatic cancer.

As current chemotherapeutic regimens could not provide substantial survival benefit with a clear increase in overall survival, several new approaches to significantly improve the clinical outcome of PC are required. Thereby, four main target groups for novel compounds can be stratified: first, downstream signaling cascades [RAS, IGFR, mitogen-activated protein/extracellular signal-regulated kinase kinase (MEK), Akt, phosphatidylinositol 3-kinase (PI3K), mammalian target of rapamycin (mTOR), Notch, signal transducer and activator of transcription 3 gene (STAT3)] within the tumors cells; second, desmoplasia and stromal response [sonic hedgehog (Shh), transforming growth factor β (TGFβ) PEGPH20 hyaluronidase]; third, tumor microenvironment and immune response [CD40L, cytotoxic T-lymphocyte-associated antigen 4 (CTLA4) antibodies, L10-interleukin-2 (IL-2) fusion product]; and fourth, vasculature and angiogenesis (153) (Figure 1).

### Signal-transduction pathways

#### The Ras, MAP2K, and MEK pathway

Activated *K-Ras*, mutated mainly at codon 12 but also less frequent at codons 13 and 61, is found in a high percentage of PC cases (154, 155). Oncogenic *K-Ras* is known to be involved in the initiation or early phase of pancreatic tumorigenesis. *K-Ras*, a member of the Ras family of genes, encodes membrane-bound GTP-binding proteins and gets activated by signaling partners, such as the epidermal growth factor receptor (EGFR). Mutation in *K-Ras* locks Ras proteins in an activated state, resulting in a continuous induction of downstream signaling cascades, such as the rapidly accelerated fibrosarcoma (Raf), MAP2K, mitogen-activated protein kinases (MAPK), and the PI3K–Akt cascades. Consequently, a variety of cellular processes, including transcription, translation, cell cycle progression, cell survival, and motility, are triggered. Several treatment approaches (156–158) targeting the Ras pathway seem to be promising for reaching a favorable outcome. Moreover, new molecules targeting downstream kinases of mutant *K-Ras* are currently investigated to overcome *K-Ras* induced drug resistance: MAP2K, though its inhibition has been only partly effective in PC therapy (159, 160) or MEK, known as one of the most significant downstream kinase of *K-Ras* signaling. As MEK mediates *K-Ras* induced effects on proliferation and survival, its inhibition might represent an innovative therapeutic target especially in tumors carrying an activating *K-Ras* mutation. A variety of small molecule inhibitors developed and validated *in vitro* for their therapeutic efficiency are under current investigation in clinical trials.

#### The EGFR, VEGFR, and IGFR-1 pathway

Tyrosine kinase receptors such as EGFR and vascular endothelial growth factor receptor (VEGFR) stimulated upon binding EGF and VEGF promote cell proliferation, survival, and angiogenesis. Therapeutic approaches inhibiting EGFR and VEGFR with monoclonal antibodies and small molecule inhibitors have been evaluated and approved for clinical use in several solid tumors (161). EGFR, a transmembrane receptor tyrosine kinase of the ErbB family, homo- or hetero-dimerizes with other ErB family members upon binding to its ligands. Thereby, a phosphorylation of tyrosine residues in its intracellular domain occurs, leading to recruitment of intracellular proteins causing downstream signaling events through Ras/Raf/MEK/MAPK, PI3K–AKT, and the STAT family of proteins. However, EGFR can be inappropriately activated due to its overexpression, activating mutations, overexpression of receptor ligands, or loss of their negative regulatory pathways. Importantly, overexpression of EGFR, which occurs in approximately 90% of PC (162) and its ligands EGF are both frequently observed in PC (163, 164). Inhibition of EGFR with the small molecule inhibitor erlotinib was in 2005 the first and until now the only Food and Drug Administration (FDA) approved targeted therapy for clinical use, providing only small survival benefit (165). The disappointing results are intriguing: EGFR signals upstream of *K-Ras* and hence, its inhibition should have almost no effect on downstream *K-Ras* driven oncogenic signals. Therefore, it is speculated, that the frequent activating *K-Ras* mutation acting downstream of EGFR accounts for the only minor impact of this inhibitor in PC. In addition, several therapeutic approaches for VEGF (166–168) have all pointed out to be ineffective in PC. These negative results of all antiangiogenetic approaches tested so far might be due to the largely hypovascular cancer surrounding stroma. Recent reported data suggest that decreasing the stromal density by the inhibition of stromal signaling pathways, e.g., Shh, might lead to enhanced intratumoral perfusion improving drug delivery and thus the efficacy of chemotherapy (169). Although, the therapeutic approaches for EGFR and VEGF showed only marginal effects, several novel targets within the receptor tyrosine kinase signaling cascades (e.g., for the IGF1R pathway) are under current investigation. IGF1R is constitutively overexpressed in 64% of PC (170). IGFR promotes survival and antiapoptotic effects in tumor cells through both *K-Ras*-dependent and independent downstream signaling cascades including the PI3K–Akt, MAPK, and STAT pathways, thus providing a promising new drug target in *K-Ras* mutated tumors (171, 172).

#### PI3K/Akt signaling pathway

Phosphatidylinositol 3-kinase/Akt signaling pathway, which is involved in cell proliferation, survival, resistance to apoptosis, angiogenesis, and invasion, represents another significant therapeutic target in PC (173). Signaling through receptor tyrosine kinases such as EGFR/IGF1R activate PI3K, which in turn activates Akt, thereby inducing multiple downstream targets, including mTOR and the transcription factor NFκB. Activation of the PI3K/AKT signaling pathway is found in 59% of PC (174) representing an independent negative prognostic factor in PC (175). Additionally, abnormal expression of the phosphatase and tensin homolog (PTEN), which normally inactivates PI3K, is frequently found in PC (176) – just as the overexpression of high mobility group A1 (HMGA1), an architectural transcription factor (177). Both alterations activate PI3K–Akt signaling, most likely responsible for the resistance to gemcitabine (178) and respective to HMGA1 suggested as promising target for therapeutic intervention (179, 180). More recently, cell-autonomous PI3K and 3-phosphoinositide-dependent protein kinase 1 (PDK1), but not Craf, were found to be key effectors of oncogenic *K-Ras* specifically in pancreas, suggesting PI3K/PDK1 as a new target for therapeutic approaches (181). Currently, therapeutic interventions targeting simultaneous inhibition of MEK/extracellular signal-regulated kinase (ERK) and PI3K/AKT signaling with the aim to overcome drug resistance mediated by upstream mutations of Ras and/or Raf are tested in advanced PC carrying K-Ras, N-Ras, and/or B-Raf mutations.

#### mTOR signaling pathway

Mammalian target of rapamycin, a serine/threonine kinase like Akt, activated by PI3K/Akt signaling and capable of regulating gene transcription and cell proliferation, represents another potential promising target. However, using mTOR inhibitors monotherapy provided only marginal benefit in patients with gemcitabine-refractory metastatic PC (182). *In vitro* studies showed evidence, that prolonged exposure of PC cells to mTOR inhibitors promote insulin receptor substrate-PI3K interactions, thereby inducing a paradoxically enhanced Akt phosphorylation and cyclin D1 expression (183). Based on these results, trials testing the effect of mTOR inhibition in combination with EGFR inhibitors were initiated and are currently ongoing. Intriguingly, the antidiabetic drug, metformin, is capable to inhibit the mTOR pathway by activating the AMP-activated protein kinase (AMPK) that negatively regulates mTOR activity via phosphorylation and stabilization of the tumor suppressor gene TSC2 (184). Recent, studies, suggest that the use of metformin is associated with a decreased risk of developing cancers (185, 186). Based on these findings, tumor cell metabolism as a potential therapeutic approach has gained rising interest. Several preclinical studies are ongoing to define modalities for earlier detection of PC and new therapeutic targets (187) via inducing changes in cellular metabolism.

#### STAT3 signaling pathway

STAT signaling pathway plays a role in cell proliferation, survival, motility, invasion, adhesion, angiogenesis, and inflammation (188). The classical STAT tyrosine phosphorylation is mediated by the Janus kinase (JAK) family of tyrosine kinases, which in turn is activated by cytokine and growth factor receptors (189, 190). In the pancreas, STAT3 is dispensable for normal development, however, in the majority of PC a constitutive STAT3 activation due to phosphorylation of Tyr705 can be found (191–193), suggesting STAT3 as a potential therapeutic target in PC. STAT3 is required for the development of the earliest pre-malignant pancreatic lesions, acinar-to-ductal metaplasia, and pancreatic intraepithelial neoplasia (PanIN) (194). More recently, IL-6 transsignaling-dependent activation of STAT3/Socs3 was found to be required for promoting PanIN progression and pancreatic ductal adenocarcinoma (PDAC) (195). Importantly, acute inactivation of STAT3 resulted in inhibition of PC initiation as previously shown in a genetically engineered mouse model (196). The important role of STAT3 in driving PDAC progression at multiple stages of pancreatic tumorigenesis *in vivo* suggests, that a pharmacological inhibition of the JAK/Stat pathway might be a promising therapeutic target.

#### Poly (ADP-ribose) polymerase pathway

Poly (ADP-ribose) polymerase (PARP) includes a family of nuclear protein enzymes, which are involved in a variety of cellular processes mainly mediating DNA damage response and apoptosis. However, only few PC patients including FPC patients carrying BRCA mutations or other defects in homologous repair might significantly respond to PARP inhibitors in combination with DNA damaging agents (197). Currently, the PARP inhibitor olaparib in combination with cisplatin and irinotecan, and also another PARP inhibitor, veliparib, in combination with different drugs are evaluated in PC (153). A recently performed *in vitro* study demonstrated that the PARP-1 inhibitor Rucaparib acts as a chemoradiosensitizer in BRCA2-deficient and -proficient PC cells (198). Moreover, *in vitro* using human PC cells and *in vivo* using a murine model, a novel function of PARP-1 in regulating the extrinsic apoptosis machinery, and also an interference combining PARP-1 inhibitors with death receptor agonists for PC therapy was proposed (199).

#### RET proto-oncogene (rearranged during transfection) pathway

The proto-oncogene RET, encoding a receptor tyrosine kinase, together with the glial derived neurotrophic factor (GDNF) were found strongly expressed in PC (200) and significantly correlated with invasion and survival after surgical resection (201). GDNF binds to the receptor tyrosine kinase and mediates through the MAPK pathway proliferation and invasiveness in PC (202, 203). A previous study demonstrated that glucose concentration-dependent expression of GDNF and RET in human PC cells correlates with alterations of cell proliferation, suggesting GDNF and RET as hyperglycemia underlying mechanism inducing PC progression (204). Further, neuroinvasive pancreatic carcinoma were found to higher express GDNF receptors RET and GRFα1 compared with normal tissue. Treating mice systemically with pyrazolopyrimidine-1, a tyrosine kinase inhibitor targeting the RET pathway, resulted in suppressed nerve invasion toward the spinal cord and prevented paralysis in mice, suggesting RET targeted therapy as a potential therapy directed against nerve invasion in PC (205). By applying the anti-RET antibody or RET siRNA *in vitro* to human PC cells the effect of GDNF on cell invasion was abrogated confirming a RET mediated GDNF effect (200). Further, a G691S RET polymorphism correlating with enhanced invasiveness was found unregulated in some human PC cells and in 37% of primary PC, representing a somatic mutation associated with PC (200). Although substantial clinical studies are missing, RET might provide a potential target for anti-invasive therapy in PC (206).

#### Gastrin

Gastrin, a peptide hormone, secreted by G cells in the gastric antrum and duodenum, acts as a growth factor for PC (207) and is expressed together with CCK-BR (the gastrin and cholecystokinin B receptor) and its precursors in 23, 95, 55–91% of PCs, respectively (208). Recent basic research studies demonstrated that knockdown of gastrin gene expression either by stable shRNA transfection (209) in human PC cells or by transient siRNA treatment (210) in gastric cancer cells as well as siRNA treatment of human BxPC-3 PC xenografts in mice results in suppressed proliferation and enhanced apoptosis. While for the orally active inhibitor Z-360 basical research studies were promising (211) and in combination with gemcitabine well tolerated by patients with advanced PC (212), substantial clinical data are missing. Other studies using the selective CCK-BR antagonist gastrazole demonstrated an improvement in patient overall survival however with no significant benefit over 5-fluorouracilin standard therapy in PC (213). Similarly, the use of gastrimmune, an immunogen, stimulating the formation of antibodies against gastrin 17 and its precursors and thus inhibiting the gastrin pathway was not effective in a phase III trial in advanced PC (214, 215).

#### Cyclooxygenase-2

COX consists of two isoforms converting arachidonic acid into prostaglandins, which is subsequently metabolized to prostaglandin E2 (PGE2), PGF2α, PGD2, and other eicosanoids. While COX-1 is constitutively expressed in many tissues exhibiting a homeostatic role, cyclooxygenase-2 (COX-2) is regulated by growth factors, cytokines, and tumor promoters. Although COX-2 expression is up regulated in 90% of pancreatic PC its impact in PC development is complex and rather unclear (216). A variety of mitogenic signaling pathways and molecules mediating invasiveness, angiogenesis, resistance to apoptosis, immunosuppression, the production of free radicals and peroxidation of procarcinogens to carcinogens are involved (217). Studies suppressing COX-2 by using NASIDs demonstrated suppression of proliferation in PC cells and angiogensis in *in vivo* and *in vitro* models (217, 218). A variety of phase II studies using Gemcitabine plus celecoxib in advanced PC were inconsistent in findings and provided only partly survival benefit (219–221). However a phase II study in advanced PC patients combining celecoxib, gemcitabine, and irinotecan resulted next to significantly improved median survival of 13 months and a 1-year survival of 64%, in improved pain and quality of life (222). More recently, apricoxib, a novel COX-2 inhibitor in phase II clinical trials, was found to significantly enhances the efficacy of gemcitabine/erlotinib in PC and promoting vascular normalization and reversed EMT (223). A further phase III trial of gemcitabine, celecoxib, and curcumin in patients with advance inoperable PC is still ongoing.

### Immune response

Over decades, the majority of research efforts had focused on molecular pathways crucial for tumor growth and maintenance thereby largely neglecting local and systemic immune response. Only recently, accumulating evidence highlighted the importance of cancer immunity, leading to enhanced tumor resistance and progression in PC (224). Currently, immunotherapies, which stimulate host immune response culminating in extensive tumor destruction (225), are under active preclinical and clinical investigations. A hallmark of PC is a distinct peritumoral stroma and an immunosuppressive tumor microenvironment rich in inflammatory cells such as T cells, macrophages, myeloid suppressor cells, etc. These immune cells, inhibit anti-tumor immunity, and enhance tumor resistance, progression, and tumor chemotherapeutic resistance, thus providing a promising target for a variety of immunotherapies (226, 227).

#### Cytotoxic T-lymphocyte-associated antigen 4

Cytotoxic T-lymphocyte-associated antigen 4 is an inhibitory signal produced by activated T cells to regulate and limit the immune response. CTLA4 can be blocked by specific antibodies such as ipilimumab or tremelimumab resulting in sustained anti-tumor immuneresponse by activated T cells. CTLA4 antibodies were the first of this class of immunotherapeutics that achieved U.S. FDA approval. In contrast, to a previously successful trial for advanced melanoma, Ipilimumab as a single agent seems to be ineffective for the treatment of advanced PC (228).

#### CD40

Another promising target is CD40, a member of the tumor necrosis factor receptor superfamily. CD40 activation can reverse immune suppression and drive anti-tumor T cell responses by mediating the “licensing” of antigen-presenting cells (229). CD40 agonists are suggested to mediate both T cell-dependent and T cell-independent immune mechanisms of tumor regression in mice and humans. Thereby, T cell-independent mechanisms seem, particularly in PC, to be linked to the re-education of tumor-promoting macrophages and stromal involution (229, 230).

#### Immune-cytokines

Cytokines, especially IL-2, characterized as one of the most potent anti-tumor cytokines, are a potential therapeutic approach in PC. Systemic application of IL-2 due to its toxicity has failed. Local therapy on the other hand seems promising (231). To guide IL-2 to the tumor site, a tumor-selective human single-chain Fv antibody fragment L19 antibody (232) which binds with high affinity to extradomain B (ED-B) of fibronectin, one of the most tumor-selective antigens associated with neoangiogenesis and tumor growth (233, 234), can be used. The resulting L19-IL-2 fusion product might be an attractive concept to enhance therapeutic effects of IL-2 by directly conjugating IL-2 to the tumor site (232).

### Stromal reaction

A hallmark of PC is the extensive peritumoral stroma and desmoplasia consisting of a variety of cellular components such as stellate cells, activated fibroblasts, and inflammatory cells, surrounded by extracellular matrix (235, 236). The stroma represents up to 90% of the tumor volume. In the past, therapeutic approaches mainly targeted tumor cells. Only recently, intense stroma has been recognized as a barrier surrounding tumor cells (236) and was hypothesized to contribute to inefficient drug delivery and chemoresistance in PC (169, 236).

#### Sonic hedgehog pathway

Sonic hedgehog pathway is known to be one of the most dominant signaling cascades contributing to enhanced desmoplasia by affecting differentiation and motility of human pancreatic stellate cells and fibroblasts thereby, influencing tumor growth in PC (237). Activation of the Shh pathway is managed by two transmembrane proteins namely tumor-suppressor PTC1 protein and oncogenic SMO protein. SMO is normally suppressed by PTC1, however, inactivating mechanisms, such as mutation of PTC1 or the binding of hedgehog proteins to PTC1, lead to continuous SMO activation and transcriptional responses. Shh promotes additional pro-tumorigenic effects by mediating cell cycle, cell survival, angiogenesis, or interference with activated *K-Ras*. Shh is expressed in about 70% of human PC (238). Previously, the hedgehog inhibitor IPI926, binding SMO, applied in a genetic mouse model resulted in a significant depletion of tumor-associated stroma (169). However, a trial investigating hedgehog inhibitor IPI926 in patients with advanced PC was terminated due to diminished overall survival in patients on the gemcitabine plus IPI926 arm. Another therapeutic target of the Shh pathway includes the transcription factor GLI1, which can be inhibited by miRNA (239).

#### Transforming growth factor β

Another pathway involved in stromal reaction is the TGFβ-dependent signaling cascade (240). TGFβ, a cytokine secreted by epithelial, endothelial, hematopoietic and mesenchymal cells, binds, and forms a heteromeric complex with the type I and II transforming growth factor β receptor (TGFBR), consequently triggering the phosphorylation of SMAD2 and 3. Cytoplasmic SMAD2/3 proteins form a complex with SMAD4, which translocates into the nucleus and activates gene transcription. However, TGFβ can also involve Ras, PI3K, and MAPK via pathways independent of SMAD. Mutations of the TGFBR1, TGFBR2, and SMAD4 genes are found in about 1, 4, and 50% of patients with PC, respectively (241). TGFβ is known to promote pro-invasive, pro-metastatic microenvironment playing a central role in stroma production, angiogenesis, and tumor-induced immunosuppression (242, 243). Inactivating mutations in the *Smad4* gene and up regulation of the inhibitory *Smad6* and *7* genes have been found in many PCs (244). Several drugs have been applied as therapeutic approach. Importantly, the antisense oligodeoxynucleotide trabedersen AP 12009 monotherapy, specifically inhibiting TGFβ2 expression, has shown a markedly enhanced survival in PC (245).

#### Hyaluronan

However, stromal-related signaling pathways such as TGFβ or Shh are not the only therapeutic possibilities; acellular matrix components can also be targeted. Hyaluronan, a non-sulfated glycosaminoglycan is highly abundant in the extracellular matrix of PC tissues (246, 247). To this effect, PEGylated human recombinant PH20 hyaluronidase (PEGPH20) leads to re-expansion of tumor blood vessels and increased concentration of gemcitabine within the tumor, resulting in significantly reduced tumor growth and enhanced survival as previously shown in a murine study (246).

### Embryonic signaling pathways

#### Notch, Hedgehog, TGFβ, and Wntβ-catenin

Oncofetal signaling pathways are typically present during fetal development, but are also frequently reactivated in cancers enhancing tumor progression and mediating resistance to chemotherapy (248). Major signaling pathways that are genetically affected in PC (249) are Notch, Hedgehog, TGFβ, and Wntβ-catenin (249). As mentioned above, TGFβ and Hedgehog signaling pathway are mainly involved in stromal reactions. On the other hand, Wnt signaling is involved in normal embryonic development and homeostatic self-renewal of a number of adult tissues. In the setting of cancer Wnt is involved in proliferation and anti-apoptosis. Aberrant activation of this pathway, due to gain-of-function mutation of activators or loss-of-function mutation of inhibitors of Wnt signaling could result in carcinogenesis and progression. Enhanced activation of Wnt signaling is found in 65% of PC (250). Importantly, Wnt signaling is induced by the hedgehog and SMAD4 signaling pathway (251, 252), which should be considered for a combined therapeutic intervention. Wnt/β-catenin signaling in PC might also be involved in chemoresistance (253) and metastasis (254), thus representing a promising therapeutic approach. The main function of the Notch signaling appears to be maintenance of pancreatic progenitor-like cells in an undifferentiated state by promoting their survival and persistence (255), similar to its function in embryogenesis (256). The Notch ligand and its receptor are highly expressed in PC compared with normal epithelial tissue (257). Activation of Notch, similar to *K-Ras* has been implicated in development of PanINs and in the initiation, progression, and maintenance of invasive PC suggesting inhibition of Notch signaling as a promising therapeutic strategy in this malignancy (258–260).

### Epigenetic changes

#### DNA methylation and histone acetylation

Epigenetic alterations are heritable with no changes in DNA sequence, and can be reversed thus representing targets for therapeutic interventions. Several epigenetic mechanisms affecting gene expression at the chromatin level are involved in carcinogenesis and tumor progression in PC such as DNA methylation associated with gene silencing and histone acetylation associated with activation of gene transcription (261). Histone acetylation leads to a reversal of positive charge on the histones, resulting in euchromatin, a loosened chromatin structure, providing transcriptionally active DNA. Histone deacetylases (HDAC) are capable to reverse this relaxation thereby reducing transcription of genes, among them several potential tumor suppressor genes. Several members of the HDAC family are highly expressed in PC, as HDAC2 and 6, thereby enhancing resistance to apoptosis (262, 263). Aberrant DNA methylation is an important cancer hallmark and associated with gene silencing. Generally, in cancer cell DNA is considered to be hypomethylated, which is associated with genomic instability and transcription of silenced transposable sequences (264). However, CpG islands found in the promoter regions of tumor suppressor genes, commonly undergo DNA hypermethylation, resulting in gene silencing, thereby promoting tumor development and progression (264). Thus, DNA hypermethylation of tumor suppressor genes might be a promising therapeutic target.

#### Telomerase

Telomeres are located at the end of chromosomes and normally decrease with each cell division limiting the lifespan of the cells. However, in a variety of malignant neoplasms including PC telomerase is strongly activated, ensuring unlimited proliferation by adding TTAGGG repeat at the end of the chromosome. Telomerase activity was found to be present in pancreatic juice of patients with PC (265) and overexpressed in 95% of PC thus providing a reliable target in PC therapy (266). Although a phase I/II trial of GV1001, a telomerase peptide vaccine designed to prime the immune system to recognize telomerase has shown promising results (267) an interim analysis of a recently performed phase III trial in 520 people with PC showed no survival benefit. Latterly, as the previous trial failed, GV1001 is tested in a phase III trial in combination with gemcitabine and capecitabine in locally advanced and metastatic PC (268). However the results of theses trial are pending.

## Conclusion

Although the exact causes driving PC initiation and progression are still unclear this review aims to summarize most important established and modifying risk factors underlying PC development. It further elucidates signaling pathways already involved in therapeutic approaches or considerable as new therapeutic targets treating PC patients. In particular, signaling pathways mediating desmoplastic stromal response and tumor immunity, largely been neglected and attracted attention only recently, might provide promising therapeutic targets for future therapy. As current therapies failed to significantly impair PC progression and improve cancer patient survival, new therapeutic approaches, and clinical studies are strongly required.

## Conflict of Interest Statement

The authors declare that the research was conducted in the absence of any commercial or financial relationships that could be construed as a potential conflict of interest.

## Abbreviations

Akt, protein kinase B; CTLA4, cytotoxic T-lymphocyte-associated antigen 4; COX-2, cyclooxygenase-2; EGFR, epidermal growth factor receptor; ERK, extracellular signal-regulated kinase; FAMMM, familial-atypical multiple mole melanoma; FAP, familial adenomatous polyposis; FDR, first-degree relatives; FPC, familial pancreatic cancer; HDAC, histone deacetylases; HMGA1, high mobility group A1; HNPCC, hereditary non-polyposis colorectal cancer; IGFR-1, insulin-like growth factor receptor-1; IL-2, interleukin-2; JAK, Janus kinase; MAPK, mitogen-activated protein kinases; MEK, mitogen-activated protein/extracellular signal-regulated kinase kinase; mTOR, mammalian target of rapamycin; PanIN, pancreatic intraepithelial neoplasia; PARP, poly (ADP-ribose) polymerase; PC, pancreatic cancer; PCMS, pancreatic-melanoma cancer syndrome; PDAC, pancreatic ductal adenocarcinoma; PDK1, 3-phosphoinositide-dependent protein kinase 1; PI3K, phosphatidylinositol 3-kinase; PJS, Peutz–Jeghers syndrome; PTEN, phosphatase and tensin homolog; Raf, rapidly accelerated fibrosarcoma; RET proto-oncogene, rearranged during transfection; RR, relative risk; Shh, sonic hedgehog; STAT3, signal transducer and activator of transcription 3 gene; TGFβ, transforming growth factor β; TGFBR, transforming growth factor β receptor; VEGF, vascular endothelial growth factor; VEGFR, vascular endothelial growth factor receptor.
